# Complement Activation in Patients with Focal Segmental Glomerulosclerosis

**DOI:** 10.1371/journal.pone.0136558

**Published:** 2015-09-03

**Authors:** Joshua M. Thurman, Maria Wong, Brandon Renner, Ashley Frazer-Abel, Patricia C. Giclas, Melanie S. Joy, Diana Jalal, Milena K. Radeva, Jennifer Gassman, Debbie S. Gipson, Frederick Kaskel, Aaron Friedman, Howard Trachtman

**Affiliations:** 1 Department of Medicine, University of Colorado School of Medicine, Aurora, Colorado, United States of America; 2 Department of Pediatrics, National Jewish Health, Denver, Colorado, United States of America; 3 Cleveland Clinic Foundation, Cleveland, Ohio, United States of America; 4 Department of Pediatrics, CS Mott Children’s Hospital, University of Michigan, Ann Arbor, Michigan, United States of America; 5 Department of Pediatrics, Children’s Hospital at Montefiore, Albert Einstein College of Medicine, Bronx, New York, United States of America; 6 Department of Pediatrics, University of Minnesota Medical School, Minneapolis, Minnesota, United States of America; 7 Department of Pediatrics, NYU Langone Medical Center, NYU School of Medicine, New York, New York, United States of America; Radboud University Medical Center, NETHERLANDS

## Abstract

**Background:**

Recent pre-clinical studies have shown that complement activation contributes to glomerular and tubular injury in experimental FSGS. Although complement proteins are detected in the glomeruli of some patients with FSGS, it is not known whether this is due to complement activation or whether the proteins are simply trapped in sclerotic glomeruli. We measured complement activation fragments in the plasma and urine of patients with primary FSGS to determine whether complement activation is part of the disease process.

**Study Design:**

Plasma and urine samples from patients with biopsy-proven FSGS who participated in the FSGS Clinical Trial were analyzed.

**Setting and Participants:**

We identified 19 patients for whom samples were available from weeks 0, 26, 52 and 78. The results for these FSGS patients were compared to results in samples from 10 healthy controls, 10 patients with chronic kidney disease (CKD), 20 patients with vasculitis, and 23 patients with lupus nephritis.

**Outcomes:**

Longitudinal control of proteinuria and estimated glomerular filtration rate (eGFR).

**Measurements:**

Levels of the complement fragments Ba, Bb, C4a, and sC5b-9 in plasma and urine.

**Results:**

Plasma and urine Ba, C4a, sC5b-9 were significantly higher in FSGS patients at the time of diagnosis than in the control groups. Plasma Ba levels inversely correlated with the eGFR at the time of diagnosis and at the end of the study. Plasma and urine Ba levels at the end of the study positively correlated with the level of proteinuria, the primary outcome of the study.

**Limitations:**

Limited number of patients with samples from all time-points.

**Conclusions:**

The complement system is activated in patients with primary FSGS, and elevated levels of plasma Ba correlate with more severe disease. Measurement of complement fragments may identify a subset of patients in whom the complement system is activated. Further investigations are needed to confirm our findings and to determine the prognostic significance of complement activation in patients with FSGS.

## Introduction

Focal segmental glomerulosclerosis (FSGS) is an important cause of glomerular disease in children and adolescents. Nearly 50% of affected patients who fail to achieve remission of their proteinuria will progress to end stage kidney disease over a 5–10 year period. Moreover, 20–25% of patients will develop recurrent disease after receiving a kidney transplant, with a substantially higher risk in patients who have already experienced recurrent disease in a prior transplant [1]. New investigations into podocyte biology have shed light on the cause of this glomerulopathy. Mutations in podocyte proteins have been identified as monogenic causes of disease in nearly 30% of patients with steroid resistant FSGS [2]. In addition, circulating permeability factors that alter the podocyte actin cytoskeleton and stability of foot processes have been identified [3,4]. Unfortunately, there are still no uniformly effective treatments for FSGS in the native kidney.

The diagnosis of FSGS is made based on histopathological findings of segmental sclerosis and hyalinosis of the glomerular tuft with variable degrees of mesangial, endothelial and epithelial cell proliferation. Immunofluorescence studies are often negative and electron microscopy does not reveal electron dense deposits. However, a sizable percentage of patients with FSGS (up to 90% in some series) manifest segmental deposition of IgM and C3 in the sclerotic portions of the glomerular tuft [5,6]. These proteins are sometimes detected in the mesangium adjacent to areas of sclerosis and in unaffected glomeruli [7]. The significance of these deposits is uncertain, but two studies have demonstrated that complement activation within the glomerulus contributes to disease progression in an animal model of FSGS, namely adriamycin nephropathy [8,9]. Furthermore, a recent study by Strassheim et al. using the same animal model found that IgM deposits within the injured glomerulus activate the classical pathway (CP) and alternative pathway (AP) [10]. The authors also demonstrated that in biopsy samples from select patients with FSGS there was co-localization of IgM and complement activation products within the glomerulus.

Factor B is a circulating protein that is required for activation of the AP [11]. Two fragments of factor B, termed Bb and Ba, are generated during this process [12]. C4a is generated by cleavage of C4 during classical pathway activation. sC5b-9, or the terminal complement complex, is generated when complement activation proceeds fully, and this multimer is often used as a marker of ongoing complement activation [13–15]. Complement activation can occur within the glomeruli of patients with FSGS [6,10]. The presence of complement activation fragments in the plasma of patients with FSGS may reflect activation within the glomeruli, e.g. within the mesangial or subendothelial space. Alternatively in patients with proteinuria, filtered complement proteins can be activated within the urinary space [16,17].

The above findings raise the possibility that complement activation contributes to glomerular injury and sclerosis in some patients with FSGS. Therefore, we performed the following study to assess whether there is evidence of complement activation in a cohort of patients with primary FSGS who participated in the NIH-funded multicenter FSGS Clinical Trial [18]. This investigation is especially relevant in light of recent advances in therapeutics and the development of agents that can selectively inhibit the complement pathway.

## Methods

### Patient characteristics

The FSGS CT was approved by the IRB at each of the participating centers. All of the subjects provided to consent to have their samples stored in the NIDDK Biorepository. The current study was reviewed and approved by the New York University Institutional review Board and the University of Colorado Institutional Review Board. Consent from patients was not required because the samples and clinical data were deidentified and the subjects had provided authorization for use of these materials in future research studies.

The FSGS CT was a multicenter, open-label randomized clinical trial that compared the safety and efficacy of cyclosporine (CSA), the active treatment control arm, to the combination of mycophenolate mofetil and oral pulses of dexamethasone (MMF+Dexa), the experimental treatment arm. Patients were enrolled at 53 participating sites. Patients were eligible for inclusion if they had primary FSGS confirmed by a central review of the diagnostic kidney biopsy, age 2–40 years old, estimated glomerular filtration rate (eGFR) >40 ml/min/1.73 m^2^ using an age-appropriate creatinine-based formula, average urine protein:creatinine ratio (Up/c) > 1 (g:g) in two first morning samples, resistance to a standard course of corticosteroid therapy, and no prior immunosuppressive therapy within 3 months of enrollment.^19^ Genetic testing was not performed as part of this study. Patients received the test therapies, CSA or MMF+Dexa, for up to 52 weeks. All patients received prednisone 0.3 mg/kg (maximum 15 mg) orally every other day for 26 weeks and lisinopril, or losartan in those who were intolerant of the angiotensin converting enzyme inhibitor, for 78 weeks. In accordance with the trial design, subjects who failed to achieve at least a partial remission at week 26 were classified as treatment failures, and the trial intervention was discontinued [19]. Patients were followed every 6 months after discontinuation of the experimental treatment for the remainder of the study period to assess clinical well-being, renal function, and occurrence of serious life threatening events.

Patient outcomes in the FSGS CT were based upon longitudinal control of the proteinuria and the primary outcome was a six-level categorical score [18]. This outcome was defined as follows: Category 1: patients who achieved a complete remission (complete remission defined as achieving a Up/c<0.2 g/g) by week 26 that was sustained to week 52; category 2: patients who achieved a partial remission (Up/c reduced by >50% to a value <2 g/g) at week 26 and then a complete remission at week 52; category 3: patients who achieved a partial remission by week 26 that was sustained to week 52; category 4: patients who achieved a partial remission at week 26 and then had recurrence of proteinuria before week 52; category 5: patients who achieved a partial remission before week 26 and then had a recurrence of proteinuria before week 26; category 6: patients who never had a Up/c reduction of 50% and an absolute value below 2 g/g. Results of the primary and main secondary outcomes were reported previously [18].

For the current study, a subgroup of 19 patients was selected at random from the available study participants who had a full set of plasma and urine samples from weeks 0, 26, 52 and 78 (Table 1). The specimens were retrieved from the NIDDK Biorepository. All samples were analyzed for CP and AP activation. Complement activation fragments were also measured in plasma from healthy controls (n = 10 for Ba, 28 for C4a, and 19 for Bb and sC5b-9) and urine from non-proteinuric control patients (n = 6). Complement activation fragments were also measured in samples from patients with CKD (n = 10 urine and plasma), systemic lupus erythematosus (n = 8 urine/23 plasma), and anti-neutrophil cytoplasmic antibody (ANCA) vasculitis (n = 8 urine/20 plasma). The clinical characteristics for these patients, where available, are shown in supplemental table 1 (S1 Table).

**Table 1:** Patient data.

			At diagnosis					At end of study		
Patient	Gender	Age	Treatment	Up/c	eGFR	Albumin	Histologic type	Up/c	eGFR	Primary outcome (1–6)
1	M	10	CSA	1.98	184.9	2.9	NOS	2.35	264.4	4
2	F	14	CSA	2.63	164.7	3.4	NOS	0.41	150.8	3
3	F	14	CSA	4.34	171.1	3.2	NOS	0.45	144	3
4	F	37	CSA	3.28	76.5	3.6	NOS	0.75	71.7	2
5	M	12	CSA	2.85	112.2	3.6	Perihilar	0.36	108.2	4
6	M	35	CSA	1.29	56.4	3.6	NOS	0.8	47.9	6
7	M	26	CSA	2.76	83.3	3.4	Perihilar	1.46	56.7	3
8	M	27	MMF	3.94	84	3.3	Tip	1.73	77.9	4
9	F	35	MMF	2.95	95.3	3.6	Collapsing	2.77	32.8	6
10	M	37	MMF	2.58	53.8	4.0	NOS	3.56	26	6
11	M	36	MMF	2.26	127.8	3.4	Tip	0.56	149.2	3
12	F	34	MMF	2.17	64.0	3.4	Perihilar	2.75	58.4	3
13	M	30	MMF	1.04	111.2	4.6	Tip	0.21	105.9	3
14	M	15	MMF	3.15	152.3	2.8	NOS	0.07	154.9	1
15	F	14	MMF	4.76	53.7	3.4	Perihilar	1.63	44.2	3
16	M	13	MMF	15.22	87.9	1.2	Collapsing	-	-	6
17	F	13	MMF	15.31	86.6	2.2	Tip	0.06	132.8	2
18	F	26	MMF	4.07	189.1	3.8	NOS	1.64	224.2	3
19	F	12	MMF	1.04	206.3	3.7	NOS	0.06	178.6	1

Abbreviations: cyclosporine, CSA; mycophenolate mofetil, MMF; Not otherwise specified, NOS.

### Measurement of complement activation fragments

Bb, Ba, C4a, and sC5b-9 levels were measured in plasma and urine using specific enzyme linked immunosorbent assays (ELISAs). All of the assays were performed using kits from Quidel Corporation (San Diego, CA) according to the manufacturer’s instructions. Briefly, samples were collected at each participating site and shipped on ice to the Biorepository. After receipt, they were processed, aliquoted, and stored at -80°C until use. Previous studies have verified that the levels of complement activation fragments in stored plasma stored with EDTA and urine samples are stable after three years [20]. The samples were thawed on ice. The plasma samples were diluted 1:10. Urine samples were diluted 1:15. For plasma and urine Ba measurements some samples were re-tested at higher dilutions (up to 1:4000) to bring the concentration within the range of the standard curve.

### Statistical methods

Abnormal values for the complement fragments in plasma and urine were defined as values exceeding the mean±2 SD in the healthy non-proteinuric control subjects. The levels of AP activation fragments were correlated with Up/c and eGFR as continuous variables and with the categorical outcomes of the FSGS CT as defined above. Statistical comparisons were performed using GraphPad Prism 5.0 software. Unpaired student T tests were used for comparison of measurements between two groups. Multiple groups were compared using one-way analysis of variance with Tukey’s post-test. Correlations were examined by Spearman's rank method and changes over time were analyzed by linear regression. A *P* value of less than 0.05 was considered statistically significant.

## Results

### Complement activation fragments are elevated in the plasma and urine of patients with FSGS

**Fig 1:**
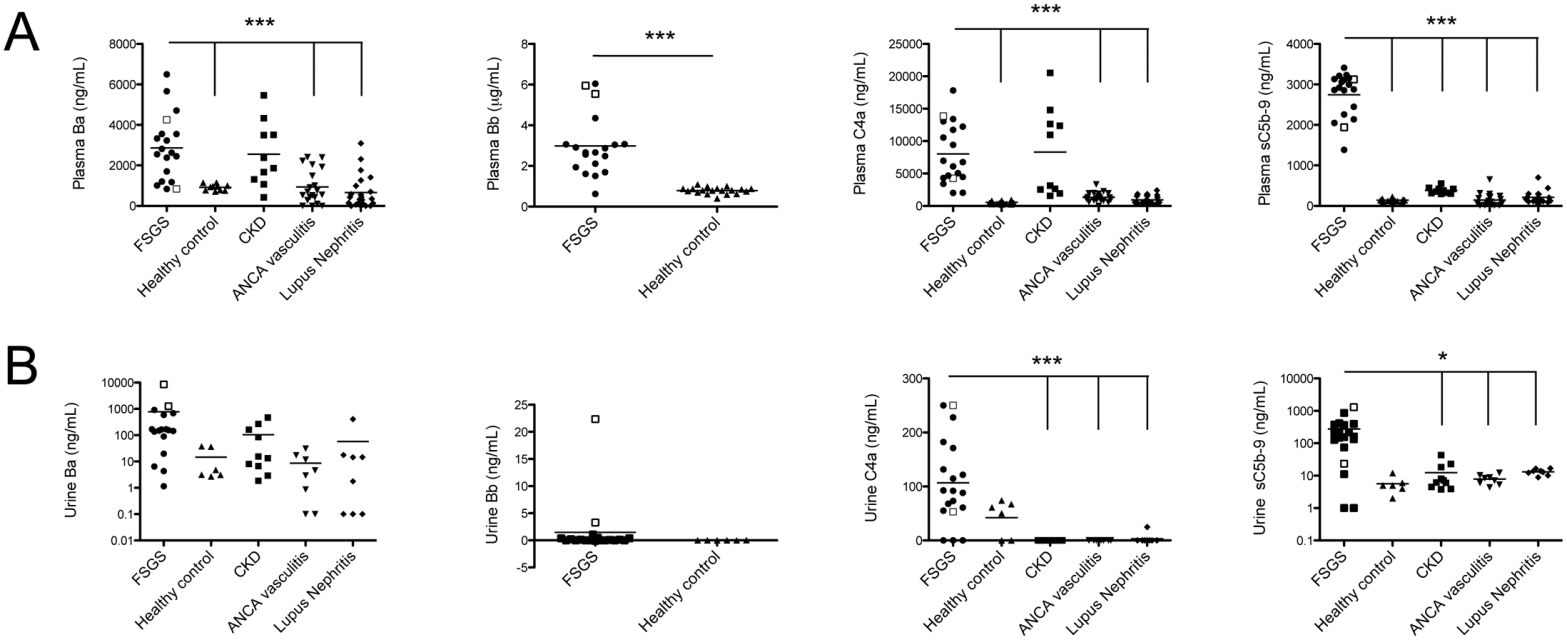
Complement activation fragments are elevated in the plasma and urine of patients with FSGS. Ba, Bb, C4a, and sC5b-9 fragments were measured in the (A) plasma and (B) urine of FSGS patients collected at the time of diagnosis. These fragments were also measured in samples from control subjects. Levels of all four complement activation fragments were increased in the plasma of FSGS patients. C4a and sC5b-9 were elevated in the urine of FSGS patients. The □ symbol indicates those FSGS patients with the full nephrotic syndrome (UPC > 3.5 g/g and serum albumin <3.0 g/dL). The groups were compared by ANOVA, and the statistical results shown are for FSGS versus the other indicated control groups. *P < 0.05, ***P < 0.001.

We measured levels of Ba, Bb, C4a, and sC5b-9 in the plasma and urine of patients with FSGS obtained at the time of diagnosis. We also evaluated plasma and urine Ba, C4a, and sC5b-9 in patients with CKD, lupus nephritis, ANCA vasculitis, and in healthy controls. Elevated levels of complement activation fragments have previously been reported in patients with lupus nephritis and ANCA vasculitis [21,22]. We found that the levels of Ba and C4a in plasma from patients with FSGS were significantly higher than levels in patients with lupus nephritis, ANCA vasculitis, and healthy controls (Fig 1). Plasma Bb was significantly higher in the FSGS patients compared to healthy controls. In addition, the differences were still highly significant when compared using the Mann-Whitney test for non-parametric data. Although the mean levels of Ba and Bb were statistically higher in the plasma of patients with FSGS, some of the individual levels for FSGS patient fell within the normal range, 5/19 (26%) and 1/19 (5%) for Ba and Bb, respectively, indicating heterogeneity within the disease. Plasma Ba and C4a levels were higher in patients with CKD than in the other groups. In addition, for 2/10 CKD patients had plasma levels of Ba were within the normal range (e.g. mean ± two standard deviations for healthy controls). Urinary levels of Ba were similar in patients with FSGS, CKD, ANCA-associated vasculitis, lupus nephritis, and healthy controls, and levels of Bb in the FSGS patients were not significantly different than those in healthy controls. Urine C4a levels were higher in FSGS patients than in ANCA-associated vasculitis, lupus nephritis, and CKD patients (Fig 1B).

The levels of plasma sC5b-9 were significantly higher in patients with FSGS than in patients with CKD, ANCA vasculitis, lupus nephritis, or in control subjects (Fig 1). Urine sC5b-9 levels were elevated in 16/19 (84%) FSGS patients, and were significantly higher in patients with FSGS than those with CKD, ANCA vasculitis, or lupus nephritis.

Levels of the complement activation fragments were compared with suPAR levels previously measured in samples from these patients, but no significant correlations were seen (S2 Fig).

### Levels of factor B fragments correlate with disease severity

**Fig 2:**
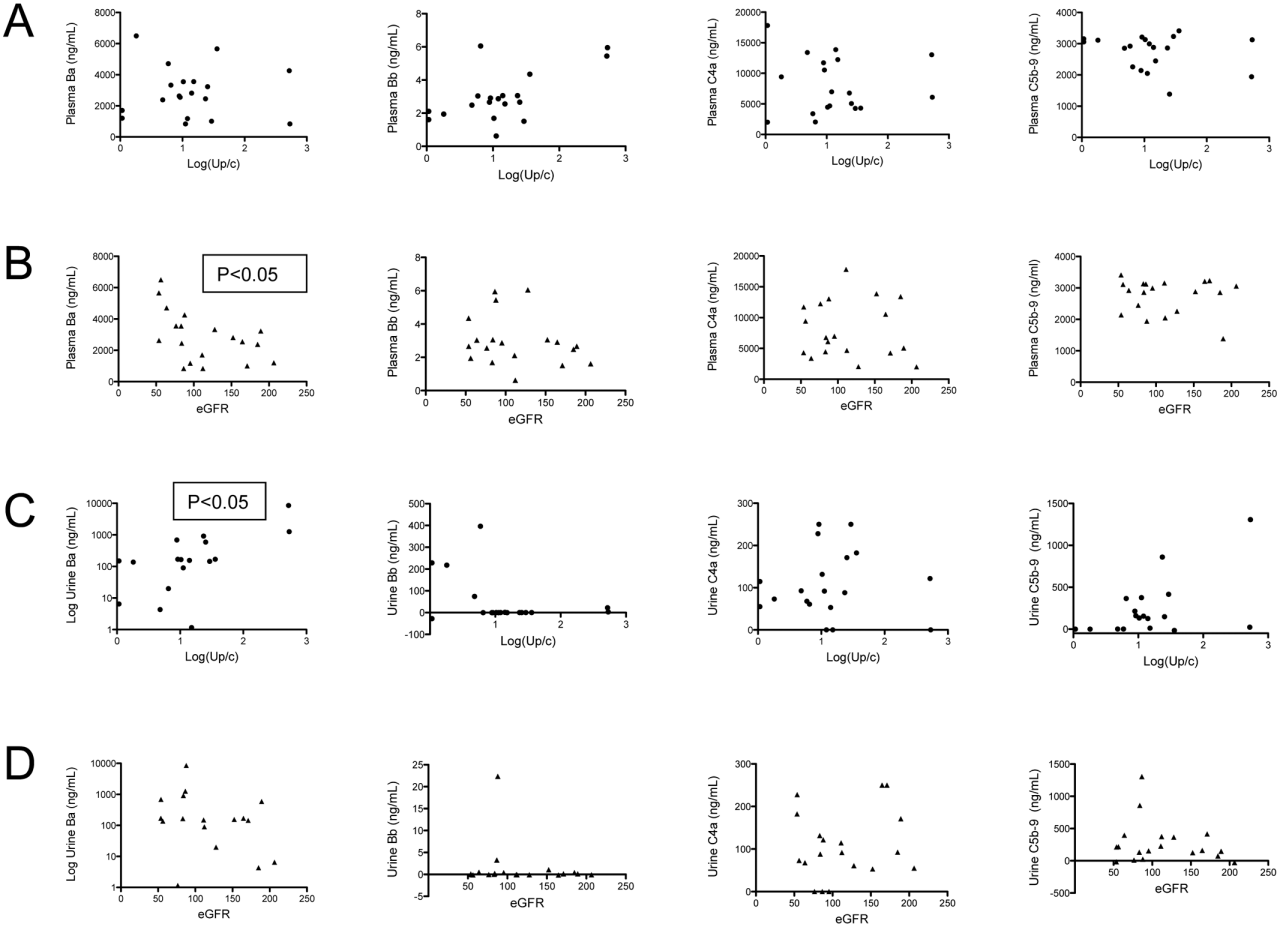
Levels of plasma Ba correlate with proteinuria and a reduced glomerular filtration rate. The levels of Ba, Bb, C4a, and sC5b-9 for individual patients were correlated with the degree of proteinuria (urine protein/creatinine ratio; Up/c) and the estimated glomerular filtration rate (eGFR). (A) None of the fragments measured in plasma were significantly correlated with the Up/c. (B) Plasma Ba was inversely correlated with the eGFR. (C) Urine Ba was significantly correlated with the Up/c. (D) None of the fragments measured in the urine were significantly correlated with the eGFR.

Plasma Ba levels were inversely correlated with the estimated glomerular filtration rate (eGFR) at the time of diagnosis (Fig 2B). An inverse correlation between the Ba level and the eGFR was also seen in pooled results for the CKD, lupus, and ANCA vasculitis control groups (S3 Fig). A wide range of Ba values were found in patients with CKD III and IV, suggesting that elevations in Ba may be due to the underlying disease and are not simply caused by a reduction in GFR. There was a significant positive correlation between urine Ba levels and Up/c (Fig 2C), although this could be due to AP activation in the glomeruli or in the urinary space. No significant correlations were seen between the levels of complement activation fragments in the urine and the eGFR (Fig 2D).

### Plasma sC5b-9 decreased in patients treated with MMF

**Fig 3:**
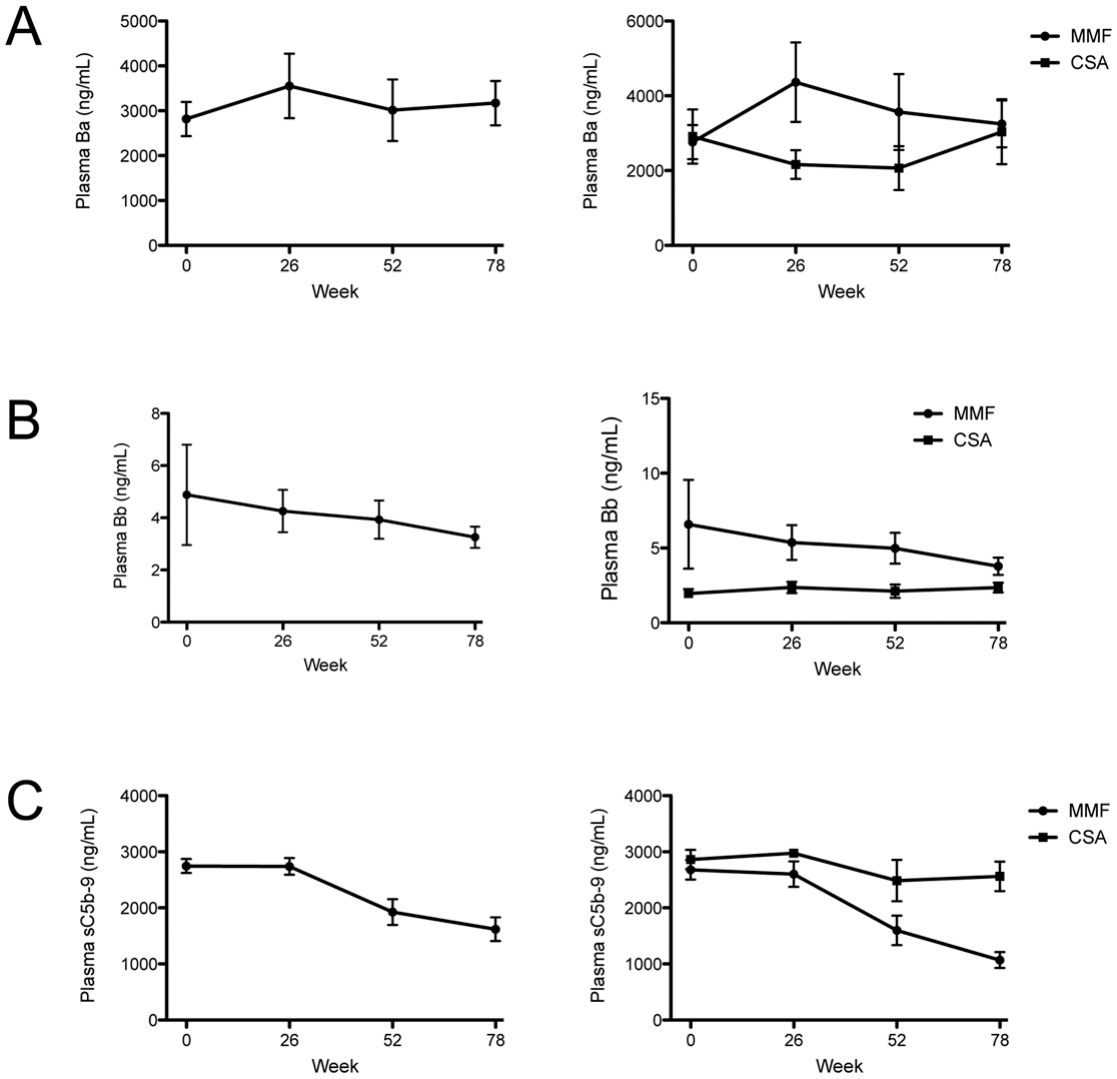
sC5b-9 patients decrease in patients treated with mycophenolate mofetil. The levels of (A) Ba and (B) Bb did not change over the course of the study (n = 19 for all time-points). (C) The levels of sC5b-9 decreased over time (P < 0.001 by linear regression; n = 19 for all time-points). When analyzed separately based upon treatment, the decrease in sC5b-9 levels was due to a decrease in patients treated with mycophenolate mofetil (P < 0.001 by linear regression; n = 12 for all time-points). The sC5b-9 levels did not decrease in patients treated with cyclosporine (n = 7 for all time-points).

Plasma sC5b-9 levels decreased over the course of the study (Fig 3). In contrast, plasma Ba and Bb levels did not significantly change. To assess the effect of treatment on generation of complement activation fragments, we examined the levels of these fragments in patients who received each treatment. The level of plasma sC5b-9 decreased in patients treated with MMF by weeks 52 and 78, but not in those treated with CSA (Fig 3C). Plasma Ba and Bb did not significantly change over the course of the study in either treatment group.

### Correlation of complement activation fragments and disease outcome

**Fig 4:**
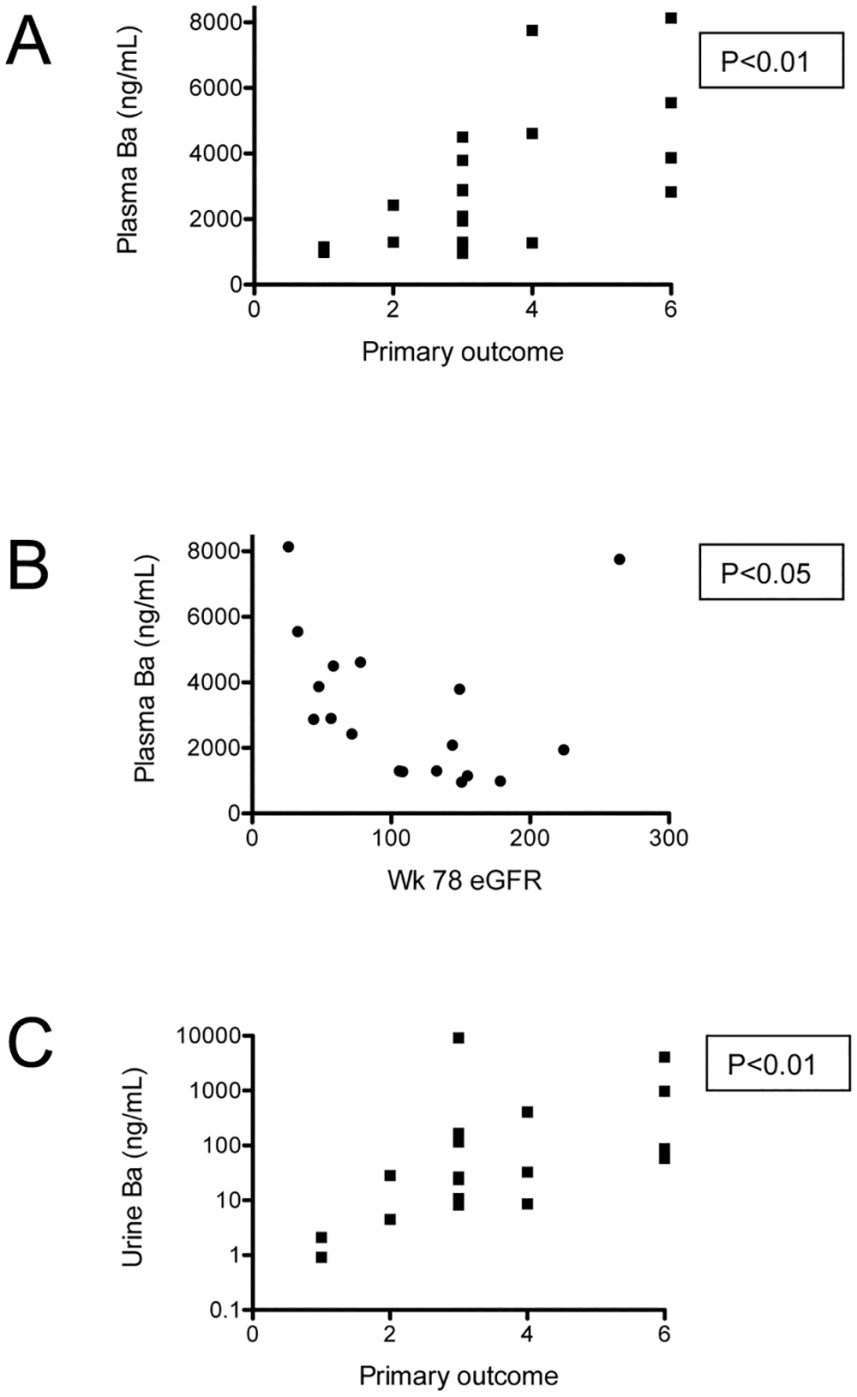
Plasma and urine Ba levels at the end of the study were correlated with clinical outcomes. Ba, Bb, and sC5b-9 levels were measured in samples obtained at the end of the FSGS CT (week 78). The plasma Ba level was significantly correlated with the (A) primary outcome and (B) eGFR at the end of the study. (C) The urine Ba level was significantly correlated with primary outcome for the study. No significant correlations between Bb levels or sC5b-9 levels and clinical outcomes were seen.

To determine whether the levels of complement activation fragments at the time of diagnosis are predictive of a patient’s disease course, we compared the level of each analyte with clinical outcomes for each patient. The primary outcome of this study was defined based on changes in proteinuria. There were no significant correlations between the levels of these activation fragments in either the plasma or urine at the time of diagnosis with the primary outcome (data not shown). However, the level of Ba in the plasma and urine at the end of the study was positively correlated with the primary outcome (Fig 4A and 4C). Plasma Ba at the end of the study was also inversely correlated with the eGFR at the end of the study (week 78) (Fig 4B).

## Discussion

It was recently shown that glomerular IgM and complement fragments co-localize in the glomeruli of patients with FSGS, and that the deposited complement proteins are in their activated form [10]. In the current study, we show that complement activation fragments are elevated in the plasma and urine of patients with FSGS. Increased levels of plasma Ba, Bb, C4a, and sC5b-9 indicate that the complement cascade is activated in a location from which the fragments can gain access to the vascular space, likely within the mesangium and regions of sclerosis. Elevations in urine Ba, C4a, and sC5b-9 in some patients, on the other hand, may reflect complement activation within the glomerulus but could also be generated by the activation of filtered proteins within the tubular lumen or lower in the urinary collecting system. Although urinary complement activation fragments may be a non-specific consequence of filtration of these proteins in patients with nephrotic syndrome [16], the detection of complement activation fragments in the plasma likely indicates that the complement system is activated in patients with primary FSGS.

In our study, the level of plasma Ba in FSGS patients was inversely correlated with the eGFR at the time of diagnosis and at the end of the study (week 78). The pooled results from the CKD, lupus, and vasculitis patients also showed an inverse correlation of plasma Ba with the eGFR. It is possible that this relationship reflects reduced clearance of Ba secondary to progressive decline in kidney function. However, nearly all of the FSGS patients had an eGFR greater than 40 ml/min/1.73 m^2^. Furthermore, plasma Ba levels in patients with CKD were not significantly higher than those in healthy controls and did not correlate with the eGFR. These results suggest an alternative explanation, namely, that increases in plasma Ba are caused by the underlying disease and are not simply due to a decline in GFR. The higher plasma sC5b-9 levels in the FSGS patients compared to all of the other groups is consistent with the proposal that there are disease-specific patterns of AP activation in patients with glomerular disease. Interestingly, Ba and C4a were elevated in patients with CKD, but sC5b-9 was not. Complement regulators such as CD59 can limit the production of downstream complement activation fragments. The elevated levels of sC5b-9 in patients with FSGS may be due to disrupted complement regulation in this disease, whereas regulation of the terminal complement cascade remains intact in CKD. We acknowledge that the range of eGFRs in the CKD comparison group was fairly narrow and it will be necessary to study a larger cohort of CKD patients to conclusively determine whether there is a relationship between a decline in GFR and an increase in plasma Ba and C4a concentrations.

Although the level of plasma Ba at enrollment did not correlate with proteinuria, Ba levels at the end of the study correlated with the primary study outcome (a clinical measure derived from changes in the level of proteinuria during the study). These observations suggest that the magnitude of AP activation is associated with disease severity. Urine Ba levels at the time of diagnosis correlated with the magnitude of proteinuria, although this could be due to activation in the urinary space, in which case it is a downstream consequence of the glomerular process. Ba and Bb are generated in equimolar amounts following activation of the AP. The discrepancy in the clinical correlations of these analytes may be due either to differences in the stability of the protein fragments, differences in the clearance of the proteins, or technical differences in the ELISAs used to detect them.

Although FSGS is not considered a “classic” immune-complex glomerulonephritis, glomerular IgM and C3 are frequently observed in areas of glomerular sclerosis, and the deposition of C4 fragments in the glomeruli of patients with FSGS has been reported [10,23]. IgM deposition is usually attributed to passive trapping of large macromolecules within the sclerotic lesion. These proteins co-localize [24], however, suggesting that the C3 deposits are due to complement activation by the IgM. Furthermore, IgM and C3 can also be observed in the mesangium of unaffected glomeruli, indicating that IgM binds to glomerular antigens in patients with FSGS [7]. In addition, although FSGS is not generally associated with frankly low levels of circulating C3 [25], the level of plasma C3 at the time of biopsy is inversely correlated with a faster rate of progression [26].

It is difficult to compare levels of complement activation products in patients with different diseases because of the varying patient characteristics (age, degree of proteinuria, etc.), and the samples from lupus and ANCA vasculitis patients that we analyzed came from patients who had started treatment. It is noteworthy, however, that that the levels of Ba, C4a, and sC5b-9 in our FSGS patients were higher than levels in these other complement-dependent diseases. It is possible that levels of the complement fragments were decreased in response to treatment of patients in those control groups, although the BVAS and SLEDAI scores indicate that many of the patients had active disease. Work in animal models of FSGS demonstrates that complement activation directly contributes to injury of the glomeruli [8–10] and tubules [9,17] in this disease. Thus, a large body of experimental and observational evidence suggests the deposition of IgM and complement proteins in the glomeruli of patients with FSGS is due to the active engagement of these components of the immune system, and that complement activation contributes to the pathogenesis of FSGS.

A major obstacle to our understanding of the pathophysiology of FSGS is the heterogeneous nature of the disease. FSGS has several histological subtypes [27]. It also can be primary or secondary to a wide range of systemic disorders, exposures, and infections. It can be genetic in nature, or it can represent a secondary adaptive disorder. Moreover, FSGS displays a variable response to treatment [28,29]. Therefore, if complement activation is a component of the pathophysiology of FSGS, it may only be involved in a subset of patients. Indeed, complement activation fragments levels were normal in some of the patients we examined, and complement proteins are only detected in the biopsies of some patients with FSGS [5,6,10]. Complement activation in patients with FSGS may be triggered by infections or other stimuli, and in susceptible patients this process continues and contributes to glomerular injury. This would be analogous to C3 glomerulopathy, in which complement activation may be triggered by infections or post-infectious stimuli but persists in patients with impaired complement regulatory function [30]. If complement activation contributes to glomerular injury, the detection of complement activation fragments in plasma and urine raises the possibility that patients in whom the complement system is activated can be readily identified and treated. Accurate biomarkers of complement activation would facilitate clinical trials of drugs aimed at inhibiting complement activation in select patients with FSGS. Future studies will explore the relationship between an initiating infection or illness and the subsequent development of FSGS.

CSA and MMF generally target the adaptive immune response. In mice, both drugs have been shown to suppress natural antibody generation [31], but their effect on natural IgM in humans is uncertain. IgM is a potent activator of the classical pathway of complement, and AP activation in FSGS may be initiated by activation of the classical pathway. Classical pathway fragments are labile and very sensitive to thawing [32], however, and we did not have samples suitable for measuring these proteins in the patients in this study. Neither CSA nor MMF would be expected to directly block complement activation. Indeed, we recently showed that CSA actually promotes endovascular complement activation through the generation of complement-activating endothelial microparticles [33]. Plasma sC5b-9 decreased over time in the FSGS patients treated with MMF+dexa, but not in the group treated with CSA. A similar discrepancy between the two treatment arms in the FSGS CT was observed when serial changes in suPAR levels were measured over the course of the study period [34]. It is possible that reduced complement activation in the glomeruli of patients treated with CSA was obscured by increased complement activation by endothelial microparticles. Nevertheless, Ba and Bb levels did not decrease over the course of the study in either treatment group, so more effective complement inhibitory drugs may be needed to suppress AP activation and improve clinical outcomes in selected patients with this disease.

We analyzed samples from only 19 patients and caution is warranted in interpreting our findings. The applicability of our findings to the broader population of patients with FSGS will need to be confirmed in future studies that involve larger patient samples. This work should be done before embarking on any interventional trial of anti-complement therapy in FSGS. This is especially the case because interference with complement activity, including the lectin pathway may be associated with an increased risk of infection. Our study is too small to draw conclusions regarding the prognostic significance of elevated complement fragments, although it is interesting that plasma Ba levels were associated with a greater degree of proteinuria and renal dysfunction. We also did not have enough patients to determine whether the degree of complement activation varies among patients with the different histological subtypes of FSGS [27] or whether it corresponds to detection of IgM and C3 within glomeruli. IgM is generally regarded as an activator of the CP, and our results suggest activation of both the CP and the AP in patients with FSGS. AP amplification can be triggered by activation of the CP, however, so it is possible that activation of both pathways is initiated by deposited IgM. It would be useful to correlate complement activation in plasma and urine with the detection of glomerular IgM and C3 within the glomeruli, and future studies will examine this question. Rituximab is reported to be beneficial in some patients with FSGS [35,36], and we have found that treatment of mice with anti-CD20 reduced glomerular IgM deposition and complement activation in adriamycin nephropathy [10]. It will also be of great interest to determine whether treatment with rituximab affects the generation of AP fragments. More work is needed to determine whether the detection of AP fragments has prognostic significance in patients with FSGS, and whether identification of patients with activation of the AP can be used to guide therapy.

In conclusion, we have demonstrated that complement activation fragments are elevated in the plasma and urine of patients with FSGS, and that complement activation in these patients likely involves the CP and the AP. These results suggest that complement proteins are deposited in the glomeruli of patients with FSGS as a consequence of activation of this innate immune system, and not due to passive trapping of the molecules in the glomerular lesions. The current study demonstrates that complement activation correlates with disease severity, but it does not establish that complement activation is a pathologic process in FSGS. A therapeutic trial with a complement inhibitory drug would be necessary to confirm whether or not complement activation contributes to disease progression. If the results of the current study are confirmed, however, the detection of complement activation fragments may enable clinicians to identify the subset of patients most likely to benefit from therapeutic complement inhibition in such a future study.

## Supporting Information

S1 Fig Correlation of complement activation fragments with serum suPAR levels. The Ba, Bb, and sC5b-9 levels in (A) plasma and (B) urine were correlated with suPAR levels measured in a previous study.^34^ No significant correlations were observed between the complement activation fragments and suPAR levels.
(TIF)

S2 Fig Plasma Ba levels correlate with eGFR in patients with other renal diseases. The Ba levels measured in plasma from patients with CKD, ANCA vasculitis, and lupus nephritis were compared to the eGFR. The plasma Ba level was significantly correlated with the eGFR for these pooled samples.
(TIF)

S1 Table Clinical characteristics of control patients. (DOCX)
