# Supplementary figures and images for: Complement Activation in Patients with Focal Segmental Glomerulosclerosis
Source: PLoS One. 2015 Sep 3;10(9):e0136558. doi: 10.1371/journal.pone.0136558 (PMC4559462; doi:10.1371/journal.pone.0136558)

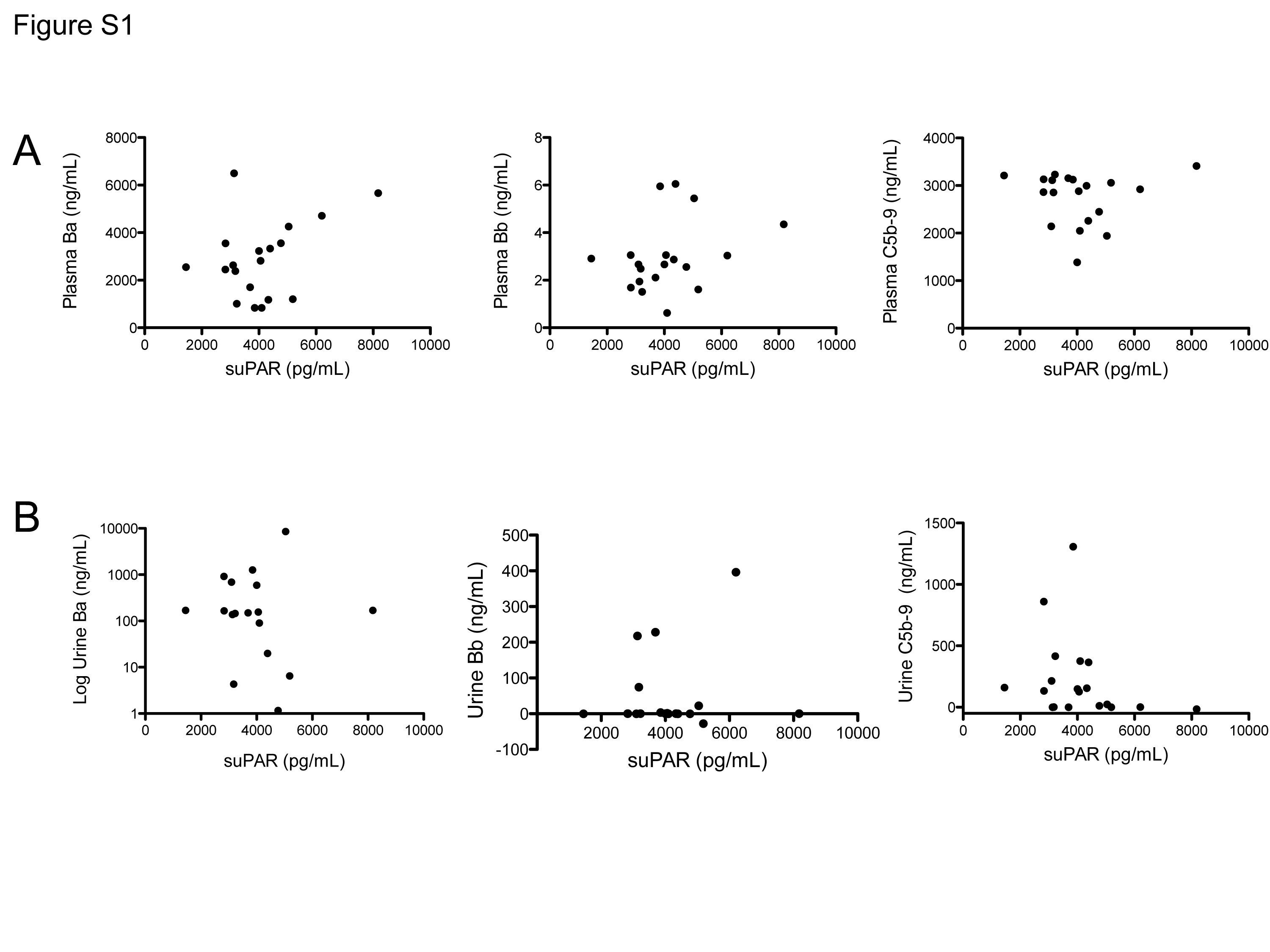

Supplement: S1 Fig — The Ba, Bb, and sC5b-9 levels in (A) plasma and (B) urine were correlated with suPAR levels measured in a previous study.34 No significant correlations were observed between the complement activation fragments and suPAR levels. (TIF) [file pone.0136558.s001.tif]

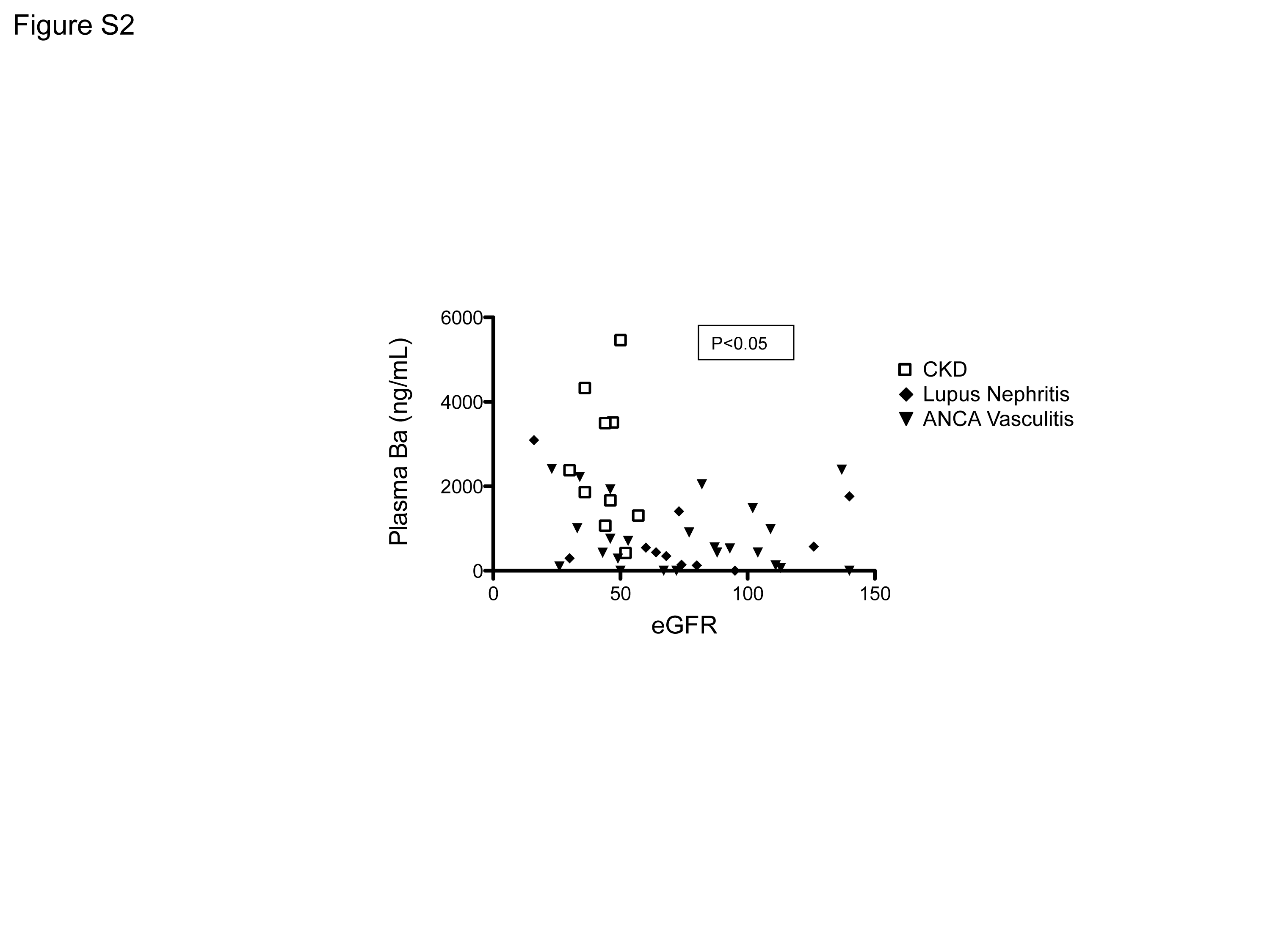

Supplement: S2 Fig — The Ba levels measured in plasma from patients with CKD, ANCA vasculitis, and lupus nephritis were compared to the eGFR. The plasma Ba level was significantly correlated with the eGFR for these pooled samples. (TIF) [file pone.0136558.s002.tif]
