# Supplementary material for: Complement Activation in Patients with Focal Segmental Glomerulosclerosis
Source: PLoS One. 2015 Sep 3;10(9):e0136558. doi: 10.1371/journal.pone.0136558 (PMC4559462; doi:10.1371/journal.pone.0136558)
Supplement: S1 Table — (DOCX) [file pone.0136558.s003.docx]

**S1 Table. Clinical characteristics of control patients.**

| **CKD** |  |  |  |  |  | |
| --- | --- | --- | --- | --- | --- | --- |
| **Patient** | **Gender** | **Age** | **eGFR** | **Albumin/creatinine ratio (mg/g)** | **Cause of renal disease** | |
| 1 | Male | 39 | 52 | 3 | Diabetes mellitus | |
| 2 | Female | 54 | 30 | 3 | Diabetes mellitus | |
| 3 | Male | 36 | 36 | 72.7 | Diabetes mellitus | |
| 4 | Male | 65 | 57 | 404 | Diabetes mellitus | |
| 5 | Male | 63 | 47 | **-** | Diabetes mellitus | |
| 6 | Male | 43 | 50 | 0.455 | FSGS | |
| 7 | Male | 62 | 44 | 10 | Polycystic kidney disease | |
| 8 | Female | 32 | 36 | 121 | IgA Nephropathy | |
| 9 | Male | 59 | 44 | 74 | Nephrolithiasis | |
| 10 | Male | 50 | 46 | **-** | GN - not otherwise specified | |
|  |  |  |  |  |  | |
| **ANCA-Associated Vasculitis** | | |  |  |  | |
| **Patient** | **Gender** | **Age** | **eGFR** | **Up/c** | **BVAS** | **Treatment** |
| 1 | Female | 47 | 88 | 0.264 | 0 | MMF |
| 2 | Male | 71 | 46 | 0.576 | 2 | MMF |
| 3 | Male | 50 | 46 | 0.074 | 1 | MMF |
| 4 | Female | 54 | 49 | 0.183 | 1 | MMF |
| 5 | Female | 56 | 72 | 0 | 6 | MMF |
| 6 | Female | 78 | 33 | 0.099 | 1 | MMF |
| 7 | Female | 50 | 87 | 1.292 | 3 | MMF |
| 8 | Male | 71 | 50 | 0.171 | 3 | MMF |
| 9 | Female | 40 | 93 | 0.277 | 3 | MMF |
| 10 | Female | 49 | 102 | 0 | 5 | MMF |
| 11 | Male | 75 | 53 | 0.083 | - | MMF |
| 12 | Female | 58 | 82 | 0.577 | - | Cyc |
| 13 | Male | 64 | 34 | 0.306 | 3 | MMF |
| 14 | Female | 61 | 23 | 1.04 | 2 | MMF |
| 15 | Female | 73 | 26 | - | 2 | MMF |
| 16 | Female | 66 | 77 | 0.239 | 0 | Cyc |
| 17 | Male | 61 | 43 | 0.338 | 2 | MMF |
| 18 | Male | 29 | 140 | 1.073 | 0 | Cyc |
|  |  |  |  |  |  | |
| **Lupus Nephritis** | |  |  |  |  | |
| **Patient** | **Gender** | **Age** | **eGFR** | **Up/c** | **SLEDAI** | **Treatment** |
| 1 | Female | 32 | 140 | 0.533 | 2 | MMF |
| 2 | Female | 32 | 133 | 1.833 | 2 | Cyc |
| 3 | Female | 36 | 73 | 0.095 | 4 | MMF |
| 4 | Female | 35 | 111 | 0.4 | 0 | MMF |
| 5 | Male | 38 | 113 | 0.128 | 0 | MMF |
| 6 | Female | 62 | 30 | 0.711 | 0 | MMF |
| 7 | Female | 26 | 126 | 0.109 | 0 | MMF |
| 8 | Female | 50 | 68 | 1.027 | 0 | Cyc |
| 9 | Female | 34 | 16 | 0.581 | 2 | MMF |
| 10 | Female | 39 | 60 | 0.172 | 0 | Cyc |
| 11 | Male | 42 | 95 | 0.209 | 0 | MMF |
| 12 | Female | 22 | 122 | 0.374 | 8 | Cyc |
| 13 | Female | 58 | 64 | 0.263 | 10 | Cyc |
| 14 | Female | 24 | 104 | 0.087 | 4 | MMF |
| 15 | Female | 40 | 109 | 3.153 | 6 | MMF |
| 16 | Male | 38 | 67 | 0.662 | 6 | Cyc |
| 17 | Female | 40 | 80 | 0.426 | - | MMF |
| Abbreviations: chronic kidney disease, CKD; eGFR, estimated glomerular filtration rate; Up/c, urine protein to creatinine ratio; BVAS, Birmingham vasculitis activity score; SLEDAI, systemic lupus erythematosus disease activity index; MMF, mycophenolate mofetil; Cyc, cyclophosphamide. | | | | | | |

**Figure legends**

**Figure 1. Complement activation fragments are elevated in the plasma and urine of patients with FSGS.** Ba, Bb, C4a, and sC5b-9 fragments were measured in the (A) plasma and (B) urine of FSGS patients collected at the time of diagnosis. These fragments were also measured in samples from control subjects. Levels of all four complement activation fragments were increased in the plasma of FSGS patients. C4a and sC5b-9 were elevated in the urine of FSGS patients. The 🞏 symbol indicates those FSGS patients with the full nephrotic syndrome (UPC > 3.5 g/g and serum albumin <3.0 g/dL). The groups were compared by ANOVA, and the statistical results shown are for FSGS versus the other indicated control groups. *P < 0.05, ***P < 0.001.

**Figure 2. Levels of plasma Ba correlate with proteinuria and a reduced glomerular filtration rate.** The levels of Ba, Bb, C4a, and sC5b-9 for individual patients were correlated with the degree of proteinuria (urine protein/creatinine ratio; Up/c) and the estimated glomerular filtration rate (eGFR). (A) None of the fragments measured in plasma were significantly correlated with the Up/c. (B) Plasma Ba was inversely correlated with the eGFR. (C) Urine Ba was significantly correlated with the Up/c. (D) None of the fragments measured in the urine were significantly correlated with the eGFR.

**Figure 3. sC5b-9 patients decrease in patients treated with mycophenolate mofetil.** The levels of (A) Ba and (B) Bb did not change over the course of the study (n = 19 for all time-points). (C) The levels of sC5b-9 decreased over time (P < 0.001 by linear regression; n = 19 for all time-points). When analyzed separately based upon treatment, the decrease in sC5b-9 levels was due to a decrease in patients treated with mycophenolate mofetil (P < 0.001 by linear regression; n = 12 for all time-points). The sC5b-9 levels did not decrease in patients treated with cyclosporine (n = 7 for all time-points).

**Figure 4. Plasma and urine Ba levels at the end of the study were correlated with clinical outcomes.** Ba, Bb, and sC5b-9 levels were measured in samples obtained at the end of the FSGS CT (week 78). The plasma Ba level was significantly correlated with the (A) primary outcome and (B) eGFR at the end of the study. (C) The urine Ba level was significantly correlated with primary outcome for the study. No significant correlations between Bb levels or sC5b-9 levels and clinical outcomes were seen.

**Figure S1. Correlation of complement activation fragments with serum suPAR levels.** The Ba, Bb, and sC5b-9 levels in (A) plasma and (B) urine were correlated with suPAR levels measured in a previous study.[^1^](#_ENREF_1) No significant correlations were observed between the complement activation fragments and suPAR levels.

**Figure S2. Plasma Ba levels correlate with eGFR in patients with other renal diseases.** The Ba levels measured in plasma from patients with CKD, ANCA vasculitis, and lupus nephritis were compared to the eGFR. The plasma Ba level was significantly correlated with the eGFR for these pooled samples.

1. Wei C, Trachtman H, Li J, et al. Circulating suPAR in two cohorts of primary FSGS. *J Am Soc Nephrol.* 2012;23(12):2051-2059.
